# APC-driven actin nucleation powers collective cell dynamics in colorectal cancer cells

**DOI:** 10.1016/j.isci.2023.106583

**Published:** 2023-04-06

**Authors:** Lautaro Baro, Asifa Islam, Hannah M. Brown, Zoë A. Bell, M. Angeles Juanes

**Affiliations:** 1 School of Health and Life Sciences, Teesside University, TS1 3BX Middlesbrough, UK; 2 National Horizons Centre, Teesside University, DL1 1HG Darlington, UK; 3 Centro de Investigación Príncipe Felipe, 46012 Valencia, Spain

**Keywords:** Cell biology, Cancer

## Abstract

Cell remodeling relies on dynamic rearrangements of cell contacts powered by the actin cytoskeleton. The tumor suppressor adenomatous polyposis coli (APC) nucleate actin filaments (F-actin) and localizes at cell junctions. Whether APC-driven actin nucleation acts in cell junction remodeling remains unknown. By combining bioimaging and genetic tools with artificial intelligence algorithms applied to colorectal cancer cell, we found that the APC-dependent actin pool contributes to sustaining levels of F-actin, as well as E-cadherin and occludin protein levels at cell junctions. Moreover, this activity preserved cell junction length and angle, as well as vertex motion and integrity. Loss of this F-actin pool led to larger cells with slow and random cell movement within a sheet. Our findings suggest that APC-driven actin nucleation promotes cell junction integrity and dynamics to facilitate collective cell remodeling and motility. This offers a new perspective to explore the relevance of APC-driven cytoskeletal function in gut morphogenesis.

## Highlights

• APC-driven actin nucleation maintains cell junction integrity and dynamics
• Normal levels of adhesive molecules require actin nucleated by APC
• Disruption of APC-driven actin nucleation impairs cell remodeling and motility
• Actin filaments nucleated by APC regulate normal cell size

## Graphical abstract

**Figure:**
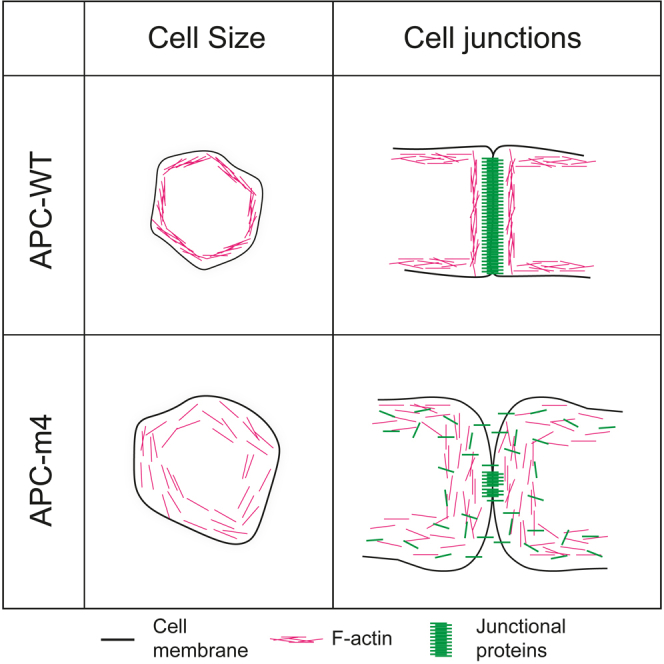

## Introduction

In the gut, cell-cell junction remodeling is key to safeguarding the integrity of the epithelial barrier and to provide structural support for the epithelial monolayers.^1^^,^^2^^,^^3^^,^^4^^,^^5^^,^^6^^,^^7^^,^^8^ In this context, the actin cytoskeleton controls the abundance of adhesive molecules at cell-cell junctions, restricts the mobility of the cell adhesion components at the membrane, and supports the integrity of cell junctions.^8^^,^^9^^,^^10^^,^^11^ Moreover, a mesh of actin is assembled under the cytoplasmic membrane that is critical for contractility of cells and their resulting architecture.^12^^,^^13^ Furthermore, actin-rich protrusions generate force for cells to migrate out of the crypts toward the villi tips where they are shed off.^14^^,^^15^

The tumor suppressor adenomatous polyposis coli (APC) is considered the master regulator of gut homeostasis.^16^^,^^17^^,^^18^ Mutations in APC, most of them sporadic, have been found in > 80% of colorectal cancer cases,^16^^,^^19^ with many leading to alterations in gut organization including the presence of (precancerous) polyps. Loss of heterozygosity via somatic mutation drives tumorigenesis. For instance, patients with inherited syndrome familial adenomatous polyposis (FAP) present hundreds of polyps formed in early adulthood with a very high risk to progress to cancer by the age of forty.^20^^,^^21^

**Figure 1:**
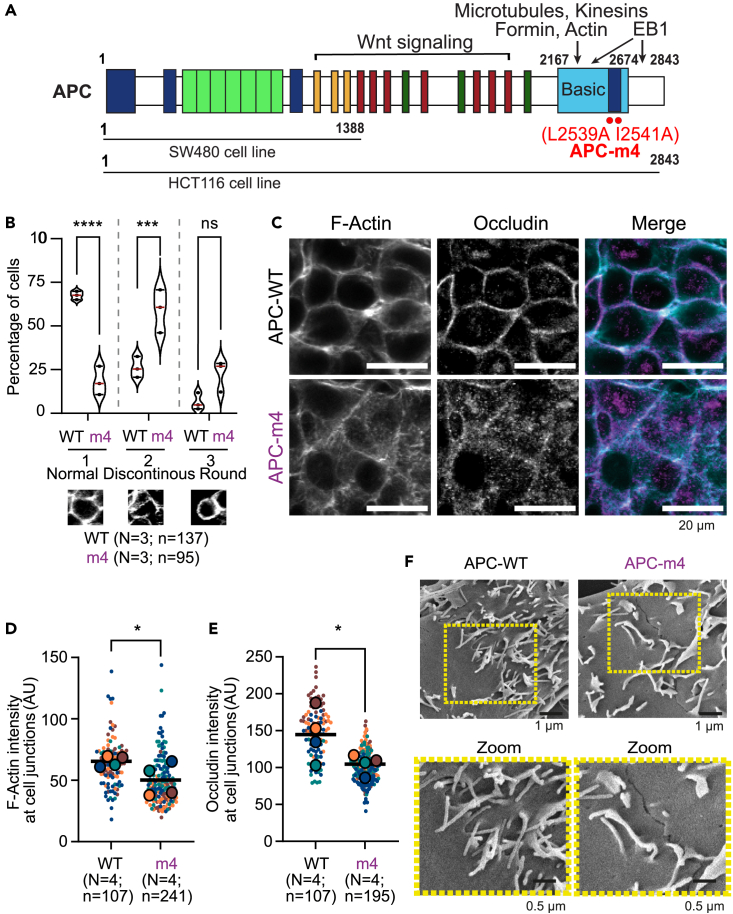
APC-m4 decreases the levels of F-actin and key molecular components at cell junctions All data are from SW480 stably expressing APC-WT or APC-m4. (A) Schematic showing the position of APC domains (colored boxes, from left to right): Dark blue boxes represent oligomerization domains, green boxes are the armadillo repeats, yellow boxes are 15 and 20 amino acid repeats, brown boxes are serine-alanine-methionine-proline (SAMP) motifs, dark green boxes are nuclear localization signal motifs (NLS), light blue box shows the basic domain (that contains one oligomerization domain shown in blue). Above the cartoon, some APC partners to the corresponding interaction sites have been stated. Red dots indicate the location of the APC-m4 mutation (L2539A I2541A) in the oligomerization motif within the basic domain. The lines below the schematic represent the two cell lines used in this study, and the corresponding length of the APC protein that they express. These are SW480 cell line, which expresses a truncated product lacking both signaling and cytoskeletal functions (1–1388 amino acid), and HCT116 cell line that expresses full-length APC (1–2843 amino acid). (B) Qualitative graph showing the percentage of cells that belong to the “normal”, “discontinuous”, and “round” category assigned in function of the appearance of the F-actin label at the cell junctions (see STAR Methods). two-way ANOVA Sidak’s multiple comparisons test was performed to find the statistical differences. “∗∗∗∗” is p < 0.0001, “∗∗∗” is p < 0.001, and “ns” is not significant. N = 3 independent repeats for each condition; n(APC-WT) = 137 cells in total, n(APC-m4) = 95 cells in total. (C) Representative immunofluorescence images of F-actin and occludin. Scale bar = 20 μm. (D) Violin plot showing the average F-actin intensity at the cell junctions. N = 4 independent repeats for each condition; n(APC-WT) = 107 junctions in total, n(APC-m4) = 241 junctions in total. (E) Violin plot showing the average occludin intensity at the cell junctions. N = 4 independent replicates for each condition; n(APC-WT) = 107 junctions in total, n(APC-m4) = 195 junctions in total. Data in D and E are displayed as “superplots” showing the mean of the different replicates (circles) and the distribution of “n junctions analyzed” (color-coded dots) was superimposed as violin plot. Solid line is the mean and paired two-tailed t test was used to find the statistical differences using N = 4 replicates. “∗” is p < 0.05. (F) Representative scanning electron microscopy images of the cell junctions. Scale bar = 1 μm, inset = 0.5 μm. See also Figure S1.

Much of our current knowledge on the roles of APC has been inferred from studies using gene silencing, knock-outs, or expression of truncated proteins lacking the C-terminal part of APC. Yet, APC is a multi-functional 2843-amino acid protein with multiple interacting partners. As such, the full biological significance of APC’s broad range of activities is far from clear. Historically, APC’s cytoskeletal roles have been overlooked,^22^ although they might play significant functions in organizing the gut epithelium, independent of APC’s control of cell proliferation via Wnt signaling.^23^ We have previously generated a specific “separation-of-function” APC mutant—herein named APC-m4.^24^ APC-m4 only changes two amino acids—L2539A I2541A—out of the 2843 residues of the entire protein (Figure 1A;^24^). APC-m4 is abrogated in F-actin nucleation but capable of binding actin monomers.^24^ Transient transfection of APC-m4 in U2OS osteosarcoma and MDA-MB231 breast cancer cells caused a delay in focal adhesion turnover and migration of single cells.^24^^,^^25^^,^^26^Evidence showed that full-length APC is required for proper localization of tight and adherens components at cell junctions, an association dependent on an “active” actin cytoskeleton.^27^^,^^28^^,^^29^^,^^30^ SW480 colorectal cancer cell line expresses a truncated version of APC (1–1388 amino acids), and therefore lacks the C-terminal domain of the protein, where the cytoskeleton activity resides. Reintroduction of full-length APC protein in SW480 rescues the localization of adhesive components and F-actin at adherens junctions, strengthening cell adhesion with concomitant changes in cell morphology and motility as assessed by a wound assay.^27^^,^^31^

These precedents raise the question of whether APC-driven actin nucleation activity may contribute to any collective cell remodeling event at cell junctions and consequent motility-associated event(s) critical for gut homeostasis. Here, we have implemented a combination of genetics, cell imaging tools, and artificial intelligence algorithms applied to the study of colorectal cancer cell monolayers to investigate the impact of APC-driven actin nucleation on a) the levels and dynamics of cell adhesion components, b) cell size, and c) the directionality of cell migration within a sheet. Our data establish a role for APC in coordinating actin with cell adhesion dynamics to control collective cell remodeling and directed cell motility in colorectal cancer.

## Results

### Actin nucleation mediated by APC is required for maintaining proper levels and dynamics of adhesive proteins at cell junctions

We have explored whether APC-driven actin nucleation activity may contribute to any collective cell remodeling event at cell junctions. To that end, we have generated colorectal (adeno) carcinoma-derived epithelial cellular lines (SW480 and HCT116) expressing stably wild-type APC (APC-WT) or the mutant APC-m4 (Figures 1A and S1A–S1D; see STAR Methods). As mentioned, SW480 cell line expresses a truncated version of APC that lacks the C-terminal part of protein, where the cytoskeleton activity resides.^32^ In contrast, HCT116 cell line expresses full-length APC.^32^ Despite effects of full length APC-m4 mutant appear to be dominant,^24^ similar to the cancer-linked C-terminal truncations of APC,^33^ we used SW480 as a control for specificity of effects related to C-terminal activities. We used these newly generated stable cell lines expressing ectopic (full length) APC-WT or APC-m4 to assess F-actin at cell-cell junctions. To do so, we first grew SW480 expressing stably APC-WT and APC-m4 epithelial monolayers, fixed and stained cells with Alexa 488-phalloidin. Confocal laser scanning microscopy showed that in SW480 APC-WT cells F-actin localized at cell junctions, as expected (Figures 1B and 1C). In contrast, in APC-m4 cells, F-actin was more diffused at cell junctions (Figures 1B and 1C). In addition, the label at APC-WT cell junctions appeared smooth and continuous compared to puncta marking cell junctions in APC-m4 cells. Based on the appearance of F-actin label at cell junctions that we correlated with their healthiness and morphology of the cell, we divided cells into three groups: “*normal*”, “*discontinuous*”, and “*round*”. From this qualitative analysis, we found that 67.49% of APC-WT cells displayed “normal” F-actin at the junctions compared to 18.24% of APC-m4 cells, 26.16% of APC-WT cells presented “discontinuous label” compared to 59.20% of APC-m4 cells, and no significant differences were found for the “round” category between APC-WT and APC-m4 cells (Figure 1B). A similar distribution of F-actin label, and therefore distribution of “*normal*”, “*discontinuous*”, and “*round*” cells was observed in HCT116 APC-WT and APC-m4 monolayers (Figure S1E). Together, these findings suggest that the loss of APC-driven actin nucleation significantly compromised F-actin localization at cell junctions in monolayers.

Subsequently, we asked whether the F-actin pool generated by APC had any effect on the levels of F-actin at cell junctions, and/or levels and dynamics of adhesive components at cell junctions. To answer those questions, we stained SW480 APC-WT or APC-m4 monolayers to visualize phalloidin (F-actin marker) along with antibodies to visualize occludin (a classic tight junction marker) by immunofluorescence. Dual-color imaging showed that F-actin and occludin localized at cell junctions in APC-WT cells as expected (Figure 1C). However, both labels were spread into the cytoplasm in APC-m4 cells (Figure 1C). Quantitative analysis showed a significant decrease in the fluorescence intensity of F-actin and occludin; specifically, at the cell-cell junctions of APC-m4 cells compared to the same labels in APC-WT cells (Figures 1D and 1E). Our results support a role for F-actin nucleation by APC in stabilizing a pool of actin and junctional proteins at cell junctions.

Next, we sought to further investigate whether APC-mediated actin nucleation affected the ultrastructure of cell junctions using scanning electron microscopy (SEM). We found that APC-WT cell attachments were well-formed and presented interdigitated junctions (Figure 1F). Recent studies have argued that the formation of membrane protrusions engaging in the form of a “handshake” or “tethering nanotubes” (known as TENT) is critical to maintain cohesive junctions.^7^^,^^34^ Accordingly, we observed prominent TENT structures in APC-WT cell junctions (Figure 1F). In sharp contrast, no interdigitation between cell junctions and barely any TENTs were observed in the APC-m4 SEM images (Figure 1F). These results confirmed that the structural connections between cells were strongly perturbed in the mutant (Figure 1F).

**Figure 2:**
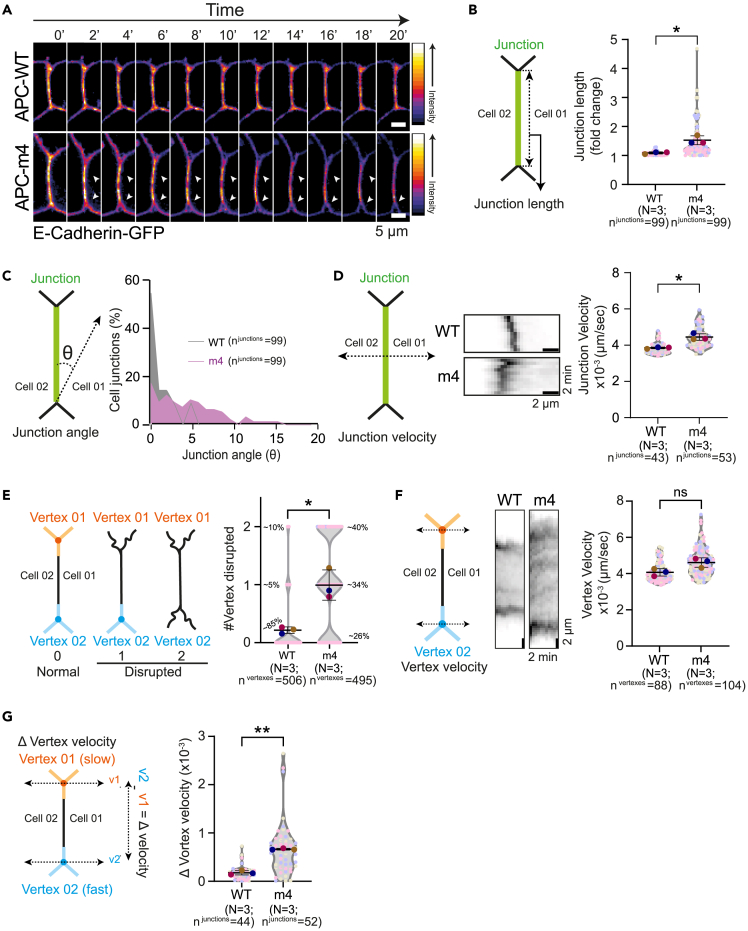
APC-m4 perturbs cell junction dynamics of epithelial monolayers All data are from SW480 stably expressing APC-WT or APC-m4 and transiently transfected with E-cadherin-GFP. (A) Representative time lapse images showing E-cadherin-GFP at cell junctions during a 20-min observation window. Scale bar = 5 μm. The color gradient shows the varying signal intensity. White arrows show disrupted cell junction vertexes. (B) Cartoon representing junction length considered for analysis. Next, violin plot showing fold change in cell junction length during the 20 min observation window, taking as a reference the time 0 min. N = 3 independent repeats for each condition; junctions: n = 9 per condition for the 11 planes of the time lapse, being that n = 99 junctions total per condition. (C) Cartoon representing junction angle considered for analysis. Next, a graph showing the percentage of cell junctions with junction angles between 0° and 20°. Cell junction angle at time 0 = 0°. N = 3 independent repeats for each condition; n = 99 junctions total per condition. (D) Cartoon representing junction velocity considered for analysis. Next, representative kymographs of cell junctions and violin plot showing cell junction velocity. N = 3 independent repeats for each condition; n(APC-WT) = 43 junctions, n(APC-m4) = 53 junctions. (E) Cartoon representing criteria of normal and disrupted junctions considered for analysis. Next, violin plot showing cells with no (0), one (1) or both (2) junction vertexes disrupted. N = 3 independent repeats for each condition; n(APC-WT) = 506 vertexes in total, n(APC-m4) = 495 vertexes in total. (F) Cartoon representing junction velocity considered for analysis. Next, representative kymographs of vertexes of cell junctions and violin plot showing vertex velocity. N = 3 independent repeats for each condition; n(APC-WT) = 88 vertexes in total, n(APC-m4) = 104 vertexes in total. (G) Cartoon representing how the difference between the velocities of two vertexes at the same cell junction was analyzed and violin plot showing those differences in vertex velocities within a junction. N = 3 independent repeats for each condition; n(APC-WT) = 44 junctions in total, n(APC-m4) = 52 junctions in total. Data in B and D-F are displayed as “superplots” showing the mean of the different replicates (circles) and the distribution of “n” junctions or vertexes analyzed, as stated in each graph, (color-coded dots) was superimposed as violin plot. Black lines are the mean and standard deviation; paired two-tailed t test was performed to find the statistical differences, except for B that ratio paired two-tailed t test was performed, using N = 3 independent replicates for all graphs. “∗” is p < 0.05, “ns” is not significant. See also Video S1.

To examine whether the loss of actin nucleated by APC had any effect on cell junction dynamics, we transiently transfected SW480 APC-WT and APC-m4 with E-cadherin protein fused to a GFP-tag—a classic marker for adherens junctions. Using live imaging in a confocal laser scanner microscope, we analyzed various junctional parameters in monolayers (Figure 2, Video S1). APC-WT cells had straighter junctions that stayed almost unchanged in length over the 20-min observation window (Figures 2A–2C). In contrast, a significant variation in both junction length and angle were observed in APC-m4 cells (Figures 2A–2C). Additionally, we quantified the movement of each cell junction over time by drawing a perpendicular line to each junction using this region of interest to acquire kymographs (Figure 2D). Junction velocity was determined from the kymograph slope. We found that APC-m4 junctions had a higher velocity than APC-WT (∼3.7 x10^−3^ vs. ∼4.3 x10^−3^ μm/s, Figure 2D). Together these data indicated that the loss of F-actin nucleation by APC led to greater plasticity of cell junctions.

Video S1. Representative time lapse showing dynamics of cell junction labeled with E-cadherin-GFP in SW480 cells stably expressing APC-WT or APC-m4, related to Figure 2 Cells were imaged every 2 min for 20 min. Video playback is 4 frames per second. Scale bar = 5 μm.

From our time lapses, we noticed that cell junctions, but especially the tricellular adhesive sites (herein named vertexes) at cell junctions exhibited less E-cadherin in APC-m4 cells relative to APC-WT cells (Figure 2A). Given that recent evidence showed that changes in E-cadherin levels at the vertexes of cell junctions affect their motion and generate an asymmetry of vertex contraction,^35^ we investigated various vertex parameters in APC-m4. We observed that vertexes were more disrupted in the mutant cells over time. Analysis of vertex integrity revealed that ∼26% of APC-m4 junctions had intact junction vertexes in contrast to ∼85% of APC-WT (Figure 2E). Moreover, instances of single or both vertexes being disrupted were ∼34% and ∼40%, respectively, in APC-m4, compared to only ∼10% and ∼5%, respectively, in APC-WT (Figure 2E). In addition, vertex velocities in APC-m4 junctions were slightly higher than in APC-WT junctions (∼4.1 x10^−3^ vs. ∼4.6 x10^−3^ μm/s; Figure 2F). Furthermore, both APC-WT vertexes in a junction had similar velocities (Figure 2G). However, APC-m4 vertexes in a junction moved at significantly different velocities (∼6.6 x10^−4^ μm/s difference; Figure 2G). Together, these data demonstrate that cells expressing APC-m4 presented lower levels of adhesive components, developed less stable and robust junctions with vertexes displaying different strength and motility compared to the junctions and corresponding vertexes of cells expressing APC-WT as control.

These data suggest that APC-driven actin nucleation is the key to preserving robust integrity and dynamics of the components at cell junctions, as well as facilitating tight structural bonds between cells.

### Loss of APC-driven actin nucleation results in larger cells

**Figure 3:**
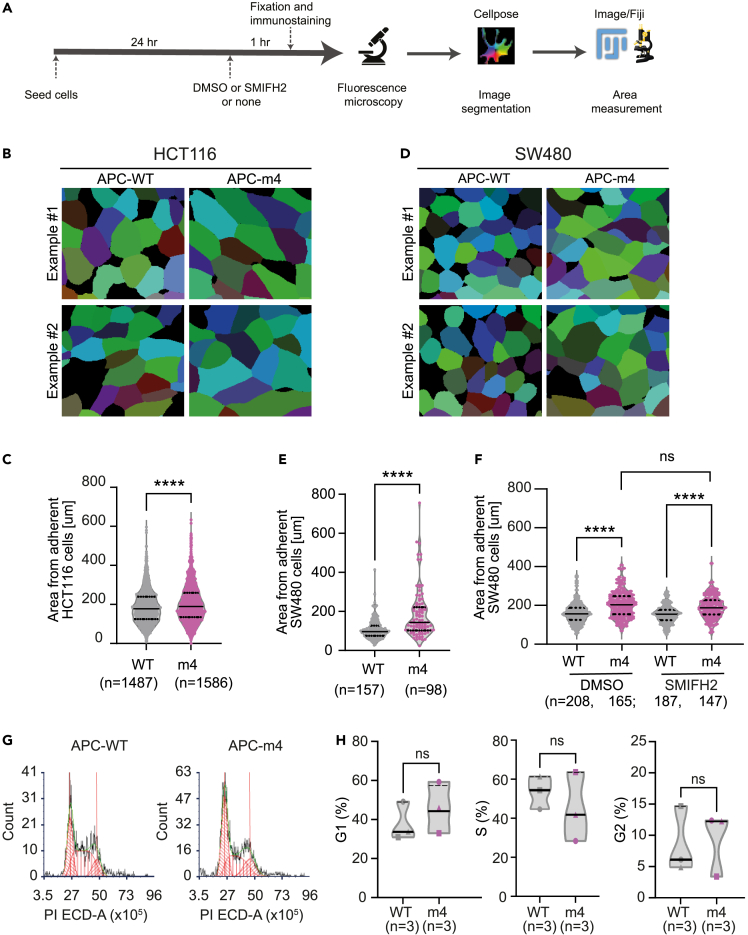
Expression of APC-m4 mutant results in enlarged cells (A) Experiment workflow. (B) Representative segmentation images from cellpose of HCT116 cells stably expressing APC-WT and APC-m4. (C) Violin plot showing the size of individual HCT116 cells shown in (B). n(APC-WT) = 1487 cells in total, n(APC-m4) = 1586 cells in total. (D) Representative segmentation images from cellpose of SW480 cells stably expressing APC-WT and APC-m4. (E) Violin plot showing the size of individual cells SW480 shown in (D). n(APC-WT) = 157 cells in total, n(APC-m4) = 98 cells in total. For panels (C) and (E), The solid line is median and dotted lines are quartiles. Mann-Whitney U test was performed to find the statistical difference. “∗∗∗∗” is p < 0.0001. (F) Violin plots showing the size of individual SW480 cells forming monolayers treated with DMSO or SMIFH2. The solid line is the median and dotted lines are quartiles. One-way ANOVA Sidak’s test was performed to find statistical differences. “∗∗∗∗” is p < 0.0001, and “ns” is not significant. n(APC-WT DMSO) = 208, n(APC-m4 DMSO) = 165, n(APC-WT SMIFH2) = 187, and n(APC-m4 SMIFH2) = 147. Data from C, E, and F graphs are from three independent repeats. (G) Representative graphs showing FACS data of HCT116 APC-WT and APC-m4 stably expressing cells. (H) From the left to the right: Violin plots showing the percentage of HCT116 APC-WT and APC-m4 cells in G1, S, and G2. N = 3 independent experimental repeats. The solid line is the median and dotted lines are quartiles. Mann-Whitney U test was performed to find the statistical difference. “ns” is not significant.

The actin cytoskeleton is central to cell shape.^3^^,^^36^^,^^37^^,^^38^^,^^39^ From our images, it seemed that some APC-m4 cells—impaired in actin nucleation—were larger than APC-WT cells. We therefore analyzed the size of individual APC-WT and APC-m4 cells grown as an adherent monolayer (Figure 3A). We used a customized version of a deep-learning-based segmentation method called cellpose.^40^ Quantification of HCT116 APC-WT and APC-m4 cells showed differences in size (187.6 μm^2^ and 203.8 μm^2^, respectively; Figures 3B and 3C). To further validate our results, we also measured the cell size in the SW480 colorectal cancer cell line in which the endogenous APC lacks the C-terminal region (where the actin-related function resides). A similar trend was found between SW480 APC-WT and APC-m4, though more pronounced than in the HCT116 APC-WT and APC-m4 cells (Figures 3D and 3E versus Figures 3B and 3C). Together, these data demonstrated that the change in cell size could be attributed to the lack of actin nucleation activity by APC.

APC also synergizes with additional nucleation proteins including formins to further promote actin filament polymerization *in vitro*.^24^^,^^41^^,^^42^^,^^43^ To determine whether formin-dependent actin polymerization contributes to cell size changes, we grew SW480 APC-WT and APC-m4 monolayers and treated them with the pan-formin small-molecule inhibitor SMIFH2 for 1 h.^44^^,^^45^ All cell monolayers were fixed, and individual cell sizes were calculated. Double inhibition of formin and APC-dependent actin activity did not result in any significant difference in cell-spread area compared to single inhibition in the mutant (Figure 3F).

To rule out that the defects observed in APC-m4 cell size were caused by changes in cell division, we performed flow cytometry analysis to determine the percentage of SW480 APC-WT or APC-m4 cells at different stages of the cell cycle (G1, M, and G2). No significant differences were found at any cell stage (Figures 3G and 3H). Overall, these results support that cell size maintenance requires proper functioning of APC-driven actin nucleation—independent of formin function and cell proliferation.

### APC-mediated actin nucleation governs collective cell migration

**Figure 4:**
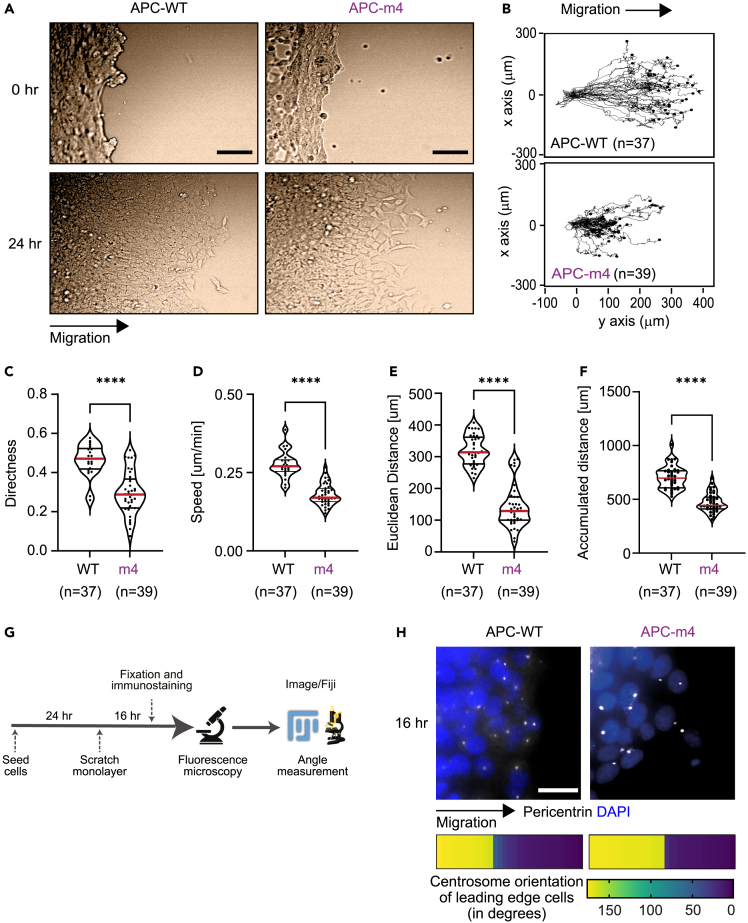
Expression of APC-m4 mutant causes defects in directionality and speed of colorectal cancer wounded monolayers All data are from HCT116 cells stably expressing APC-WT or APC-m4. (A) Representative wound healing assays showing cells at 0 and 24 h after scratch. The arrow indicates the direction of the migration front. Scale bar: 100 μm. (B) Representative traces of the migration paths of individual cells, moving into a wound site, displayed in horizontal arrays. Black arrow indicates the direction of the migrating cells. (C–F) Violin plot showing the directionality (C), velocity (D), euclidean distance (E), and accumulated distance (F) of individual cells from the edge of the wound during a 24-h observation window. The red line is the median and the black lines are the quartiles. Mann-Whitney U test was performed to find statistical differences. “∗∗∗∗” is p < 0.0001. Data in all panels are from three independent repeats. In panels B to F, n(APC-WT) = 37, n(APC-m4) = 39. (G) Experiment workflow. (H) Above, representative images of migrating cells co-stained with pericentrin antibodies (centrosome marker) and DAPI (nuclear marker) at 16 h from wounding. Below, heat maps showing the orientation of the centrosome toward the leading edge from APC-WT and APC-m4 cells treated as in (G). The color gradient shows the different angles with intensities ranging from 0 to 180 grades (in degrees) that correspond to less oriented (yellow) to more oriented (purple), respectively, toward the leading edge; as represented in the cartoon. Orientation in grades was calculated as the angle from pericentrin in relation to the nucleus and to the front of the wounding migrating cells. Mann-Whitney U test was performed to find statistical differences. “ns” is p < 0.05. Data are from three independent repeats; n(APC-WT) = 31 angles (one per cell), n(APC-m4) = 33 angles (one per cell). See also Figure S2.

Collective cell migration requires a constant modification of cell shape, which is intrinsically coupled with dynamic remodeling of the actin cytoskeleton.^46^^,^^47^^,^^48^ Therefore, we explored whether collective cell migration was compromised in APC-m4 colorectal cancer cells. To address this, HCT116 cells expressing APC-WT or APC-m4 were grown as monolayers, scratched to create a wound and imaged during healing (Figure 4A, Video S2). We analyzed the trajectories of individual cells migrating at wounds over time and quantified directionality, migration speed, accumulated traveled, and Euclidean distances (the shortest distance between two points, see STAR Methods). We observed that the APC-m4 cell trajectories were more erratic (cells moved significantly slower and covered shorter distances than the APC-WT cells) indicating random cell migration (Figures 4B–4F). In addition, we observed that mutant cells exhibited a high incidence of cell detachment (Figure 4A, Video S2), also contributing to the loss of coherent directionality. Similar results were observed in SW480 colorectal cell monolayers (Figure S2).

Video S2. Representative time lapse showing the motility of HCT116 stably expressing APC-WT or APC-m4 cells in a wound healing assay, related to Figure 4 Cells were grown to a monolayer, then wounded, and time lapsed for 24 h. Images were acquired every 30 min. Video playback is 12 frames per second. Scale bar = 100 μm.

Since APC regulates centrosome reorientation to establish polarity of cell migration,^49^^,^^50^^,^^51^ it could be that APC-dependent actin nucleation effects on migration were due to cell polarity defects. To investigate that, APC-WT and APC-m4 epithelial monolayers were grown, scratched, and after 16 h from wounding, migrating cells were fixed. All cells were co-stained with pericentrin antibodies (a marker for centrosome reorientation) and DAPI (nuclear marker) (Figure 4G). Analysis of centrosome orientation toward the cell front did not show major differences between APC-WT and APC-m4 cells, suggesting that the front-rear axis is not altered by loss of APC-driven actin nucleation (Figure 4H). These results implicate APC-driven actin nucleation in promoting proper directionality and speed of colorectal cells moving as a collection, independently of its cell polarity roles.

## Discussion

In this study, we have exploited the power of a specific “separation-of-function” APC mutant named APC-m4 that specifically abrogates actin nucleation function^24^ to probe APC’s involvement in cell-cell junction integrity via the actin cytoskeleton. Our study offers crucial mechanistic insight to bridge the gap between two functional links previously proposed: APC and cell junctions, and F-actin and cell junctions. Specifically, our findings integrate these two links by highlighting the requirement of APC-driven actin nucleation activity to sustain cell junction integrity and strength, cell size, and consequent directionality of cell migration.

It has been shown that cells expressing truncated APC proteins lacking the C-terminus exhibit reduced levels of E-cadherin molecules at cell junctions.^31^^,^^52^ In turn, the expression of full-length APC restores cadherin protein levels, suggesting a relevant role of the C-terminal domain of APC.^31^ Others proposed that a mesh of F-actin surrounds E-cadherin clusters to act as an “insulator” or “corral” to delimit their location, fusion, and mobility.^11^^,^^53^ Indeed, inhibition of formin activity reduces both F-actin and E-cadherin levels, with concomitant perturbation of E-cadherin stability at cell junctions.^54^ It is believed that rearrangements at cell junctions may be governed by forces yet to be defined in mechanistic terms. For example, actomyosin-based contractility might induce conformational changes in adhesive proteins, in turn, affecting their stability and/or binding to partners.^55^^,^^58^^,^^57^^,^^56^ Interestingly, modulation of E-cadherin levels at the vertexes of cell junctions affects their motion and generates asymmetry of vertex (and therefore cell junction) contraction.^35^

Here, we show that expression of APC-m4 in colorectal cancer cells led to a reduction in F-actin at cell junctions relative to the levels observed in cells expressing APC-WT. Accordingly, APC-m4 cells presented cell junctions with reduced levels of adhesive components, as well as perturbed dynamics, including a failure to maintain cell junction length and angle, an increase in the incidence of vertex disruption and a perturbed correlation of velocities between the two vertexes in a junction that shifted from symmetric to asymmetric vertex contraction. In support of the precedents outlined above, our data suggest that the pool of actin nucleated by APC might act as a fence that confines adhesive components within cell junctions and, at the same time, confer force to enable cell junction rearrangements while preserving their integrity and strength.

Dynamic cell shape, subject to remodeling of the actin cytoskeleton, is critical to proper cell migration.^3^^,^^36^^,^^47^^,^^59^ Interestingly, we found that APC-m4 cells were larger and moved more erratically and slowly than APC-WT cells, despite being able to polarize centrosomes toward the leading edge. The larger size of mutant cells might stem from the reduction in E-cadherin, prompting the translocation to the nucleus of the yes-associated protein 1 (YAP) transcription factor.^60^ Alternatively, but perhaps along with any potential alteration of YAP, it could be due to the reduction in actin polymerization that in turn affects cell stiffness area and volume.^59^ The fact that APC-m4 migrating cells did not present major problems in repositioning centrosomes toward the leading edge compared to APC-WT cells goes in line with previous reports. On one hand, APC’s clustering at microtubule plus-ends and consequent stabilization of microtubules at the leading edge seems to be required for the establishment of normal epithelial polarity.^23^^,^^49^^,^^50^^,^^51^^,^^61^^,^^62^ On the other hand, focal adhesion assembly or disassembly rates between focal adhesions located at the leading versus trailing edges in APC-m4 individual cells did not show any statistical differences compared to APC-WT cells.^25^ It is worth mentioning that APC-m4 mutant cells are capable to bind to G-actin monomers,^24^ and somehow APC-actin interactions could facilitate interplay between both cytoskeletons to reposition centrosomes toward the leading edge front in the mutant cells. These observations suggest that independently of APC’s roles in cell polarity and/or microtubule stability, APC-driven actin nucleation activity in colorectal cancer cells may be required for proper shape and cell-cell attachments during cell migration to sustain their directionality and speed.

### Conclusion

We propose that actin nucleated by APC acts as a “force buffer” to support the build-up of adhesive molecules allowing cells to undergo the required rearrangements, underlying correct cell junction dynamics. As a result, this actin pool might contribute to sustaining the structure of the epithelial monolayer, lining the gut, and facilitate its deformation to ultimately render the typical crypts-villi landscape. Then, APC-driven actin nucleation might be involved in the “epithelial morphology” pathway to control gut organization independently of the microtubule dynamics and Wnt signaling pathways.^23^ In addition, the effects of APC-driven actin nucleation on directed cell migration in wounded monolayers, suggest that APC alone, or perhaps in coordination with Arp2/3 and/or formins, might be critical in orchestrating directed cell motility along the crypt-villus axis.

Our results provide a further connection between the well-established relevance of actin polymerization activity for the integrity of the gut barrier and the architecture of the gut monolayer on one hand, and APC’s standing as the master regulator of gut homeostasis on the other. In our view, APC-mediated actin nucleation activity at cell junctions would not only support the integrity of the epithelial barrier that is critical to avert infection or inflammatory disorders, such as inflammatory bowel disease but also to withstand epithelial cell deformation that could otherwise drive the path to polyps and ultimately to colorectal cancer. Thus, our findings offer a new perspective to explore the relevance of APC-driven cytoskeletal functions in gut morphogenesis and human disease.

### Limitations of the study

The major limitation of this study is that as a model system, we have used cell monolayers. In such set ups, cells are not in their physiological environment and interactions with the extracellular matrix that can influence the results can be missed. Supporting assays and analysis in intestinal organoids expressing endogenous APC wild-type or mutant—which would mimic patient response in a more physiological context—would validate the effects of APC-driven actin nucleation in collective cell dynamics. However, work on organoids, especially expressing full-length APC or point mutations at endogenous levels, as well as live imaging studies to monitor cell remodeling and migration using organoids is currently very challenging. Development of those experimental models and powerful technologies would provide invaluable insights to determine the role of APC-mediated cytoskeletal activities in gut tumorigenesis.

## STAR★Methods

### Key resources table

**Table:** 

REAGENT or RESOURCE	SOURCE	IDENTIFIER
**Antibodies**		

Rabbit anti-APC	Abcam	Cat# ab154906; RRID:AB_2861396
Rabbit anti-Occludin	Abcam	RRID:AB_1661439
Mouse alpha-tubulin	Santa Cruz Biotechnology	Cat# sc-32292; RRID:AB_2110259
Rabbit anti-Pericentrin	NovusBiologicals	Cat# NB100-61071; RRID:AB_925559
Goat anti-mouse AlexaFluor-488	Thermo Fisher Scientific	Cat# A32723; RRID:AB_2633275
Goat anti-rabbit AlexaFluor-488	Thermo Fisher Scientific	Cat# A11008; RRID:AB_2284594
Goat anti-rabbit AlexaFluor-555	Thermo Fisher Scientific	Cat# A-21428; RRID:AB_2535849
Goat anti-mouse AlexaFluor-633	Thermo Fisher Scientific	Cat# A-21050; RRID:AB_2535718
Alexa-568 anti-rabbit	Invitrogen-Thermo Fisher Scientific	Cat# A-11011; RRID:AB_141416

**Chemicals, peptides, and recombinant proteins**		

AlexaFluor-568-Phalloidin	Invitrogen-Thermo Fisher Scientific	Cat# A12380
AlexaFluor-488-Phalloidin	Invitrogen-Thermo Fisher Scientific	Cat# A12379
DAPI	BD Biosciences	Cat # 564907; RRID:AB_2869624
DMEM- Dulbecco’s Modified Eagle Medium high glucose with pyruvate and L-Glutamine	Thermo Fisher Scientific	Cat# 41966029
FBS – Fetal bovine serum	Sigma-Aldrich	Cat# F9423
Antibiotics (Penicillin and Streptomycin)	ThermoFisher	Cat#15140-122
Phosphate-Buffered Saline (PBS)	Sigma-Aldrich	Cat# P4417
Phosphate-Buffered Saline (PBS)	ThermoFisher	Cat# 20012027
Lipofectamine 3000	Thermo Fisher Scientific	Cat# L3000-015
G418	Thermo Fisher Scientific	Cat#10131035
Opti-MEM reduced serum medium	Thermo Fisher Scientific	Cat# 11058021
Levo L15 (Leibovitz)	Thermo Fisher Scientific	Cat# 21083027
DMSO	Sigma-Aldrich	Cat# 276855
SMIFH2	Sigma-Aldrich	Cat# S4826
Triton X100	Sigma-Aldrich	Cat# T8787-100ML
Tween20	Sigma-Aldrich	Cat# P1379-100ML
Advanced BioMatrix - PureCol® Solution, 3 mg/ml (bovine)	AdvancedBiomatrix	Cat#5005
RNAse solution	Merck	Cat# 70856-3
Propidium Iodide solution	Merck	Cat# 25535-16-4
BSA	Sigma-Aldrich	Cat# A3059-10G
Glutaraldehyde, EM Grade 25%	Polysciences	Cat# 01909-10
Osmium tetroxide (OsO4)	Sigma-Aldrich	Cat# O5500-250MG
Hexamethyldisilazane (HMDS)	Merck Life Science Ltd	Cat# 804324
Paraformaldehyde 16% - methanol free (PFA)	Thermo Scientific	Cat# 28908

**Experimental models: Cell lines**		

HCT116 human colorectal cell line	Merck	RRID:CVCL_0291
SW480 human colorectal cell line	Merck	RRID:CVCL_0546
HCT116 stably expressing APC-WT	This work	This work
HCT116 stably expressing APC-m4	This work	This work
SW480 stably expressing APC-WT	This work	This work
SW480 stably expressing APC-m4	This work	This work

**Recombinant DNA**		

Plasmid: APC-m4	[16]	Bruce Goode-US
Plasmid APC-WT	Addgene	Cat#16507; RRID:Addgene_16507
Plasmid: GFP-alone	Addgene	Cat# 54759; RRID:Addgene_54759
Plasmid: E-cadherin-GFP	Addgene	Cat# 280009; RRID:Addgene_28009
Plasmid: Life-Actin-mCherry	Addgene	Cat# 54491; RRID:Addgene_54491

**Software and algorithms**		

LAS X Life Science Microscope Software version 3.5.5.19976	Leica	RRID:SCR_013673
Chemotaxis and Migration Tool software version 2.0	Ibidi, Germany	RRID: SCR_022708
FlowJo™ software version 10.8.1	BD Life Sciences	RRID:SCR_008520
GraphPad Prism version 9	GraphPad Software	RRID: SCR_002798
Adobe Illustrator CC version 26.5	Adobe Illustrator (Adobe Systems)	http://www.adobe.com/products/illustrator.html; RRID:SCR_010279
python version 2.12	python	RRID:SCR_008394
Microsoft Excel version 16	Office365	RRID:SCR_016137
CellPose	Stringer et al.^40^	RRID:SCR_021716
Fiji / ImageJ version 1.53k	NIH – public domain	RRID:SCR_003070
Fiji / ImageJ version 2.3	NIH – public domain	http://fiji.sc; RRID:SCR_002285
“multi-kymograph” tool from Fiji	NIH – public domain	RRID:SCR_021717

**Other**		

AquaMount mounting medium	Thermo Fisher Scientific	Cat# 14-390-5
Circular Round cover glasses 0.18 mm	VWR International Ltd UK	Cat# 631-0153
Chambers precoated with collagen IV	Ibidi	Cat# 8082
Glass Slides	Academy	Cat# N/A141

### Resource availability

#### Lead contact

Requests for further information and reagents should be directed to and will be fulfilled by the Lead Contact, Maria Angeles Juanes (majuanes@cipf.es).

#### Materials availability

This study has generated new cell lines which would be made available upon request to the lead contact Maria Angeles Juanes.

### Experimental model and subject details

HCT116 (#91091005 from Merck, RRID:CVCL_0291) and SW480 (#87092801 from Merck, RRID:CVCL_0546) human colorectal cancer cell lines were certified by short tandem repeat DNA profiling authentication and a negative test for mycoplasma contamination. Cells were grown in Dulbecco’s Modified Eagle’s Medium (DMEM high glucose with pyruvate and L-Glutamine (#41966029; ThermoFisher) supplemented with 10% Fetal Bovine Serum (FBS; # F9423; Sigma-Aldrich) and antibiotics (Penicillin and Streptomycin (#15140-122; ThermoFisher) at 37°C, 95% humidity, and 5% CO_2_.

HCT116 and SW480 colorectal cancer cell lines stably expressing APC-WT and APC-m4 were generated by transfecting each parental cell line with plasmids encoding APC-WT (#16507; Addgene) or APC-m4.^24^ A plasmid encoding GFP-alone (#54759, Addgene) was used as transfection control. After plasmid transfection, cells were grown in medium containing 600 mg/ml G418 (#10131035; ThermoFisher) until single colonies were observed. Four colonies per condition were picked, APC expression was studied using immunofluorescence assays, and cells were maintained in medium containing 200 mg/ml G418). Several works proposed that APC expression levels seem to be autoregulated, and its expression levels (and detection) depend on cell line and antibodies.^24^^,^^63^^,^^64^ In addition, expression levels of transfected APC (WT) and APC (m4) on top of endogenous and/or in APC null U2OS cells were remarkably similar to endogenous APC (Juanes et al.^24^). Hence to select clones we considered APC-WT and APC-m4 clones expressing similar APC levels than the corresponding parental cell line (containing only endogenous APC; clones expressed 1-1.25-fold APC over endogenous; as shown in Figure S1). Within clones expressing ‘ectopic’ APC-WT and APC-m4, clones with comparable APC levels were used in subsequent experiments and for a maximum of 10 passages.

### Method details

#### Plasmid transfection

Plasmid transfection was performed using Lipofectamine 3000 (#L3000-015; ThermoFisher) according to the manufacturer’s instructions. In addition to the standard protocol, after 4 hours, the transfection medium was removed and fresh pre-warmed DMEM was added to each well.

#### Immunostaining studies and drug treatments

To determine the APC protein levels in HCT116 and SW480 colorectal cancer cell lines stably expressing APC-WT and APC-m4, cells were seeded on 18 mm^2^ round coverslips (#631-0153; VWR International Ltd UK). To determine Occludin and F-actin protein levels at cell junctions, cells were seeded on 8-well chambers with collagen IV (#8082, ibidi, Germany) until confluency. To determine cell size from cell monolayers, cells were grown on round coverslips until confluency, then treated with DMSO (as control, #276855, Sigma-Aldrich) or SMIFH2 (20 μM, #S4826, Sigma-Aldrich) for one hour before proceeding for staining. All cells were fixed with warm 4% paraformaldehyde (#28908, Thermo Scientific) in Phosphate-Buffered Saline (PBS: 2.7 mM KCl, 1.8 mM KH_2_PO_4_, 10 mM Na_2_HPO_4_, 140 mM NaCl, pH 7.4) (#P4417 Sigma-Aldrich) for 15 minutes, washed once with PBS, permeabilized with 0.1% Triton X-100 (# T8787-100ML, Sigma-Aldrich) for 10 minutes, washed once with 1x PBST (1× PBS, 0.1% v/v Tween-20 # P1379-100ML; Sigma-Aldrich), blocked with 3% BSA (#A3059-10G; Sigma-Aldrich) for 1 hour at room temperature and incubated overnight at 4°C with anti-APC C-terminus (1:250, Abcam, ab154906, RRID:AB_2861396), anti-Occludin (1:300, Abcam, RRID:AB_1661439), anti-Pericentrin (1:1000, NovusBiologicals, # NB100-61071, RRID:AB_925559), accordingly. Coverslips were then washed with 1x PBST three times for 5 minutes each and incubated with secondary antibodies Alexa-488 anti-rabbit goat (1:1000; A11008, RRID:AB_2284594 Thermo Fisher Scientific) or Alexa-568 anti-rabbit (1:1000, # A-11011, Invitrogen-Thermo Fisher Scientific), along with Alexa-568 Phalloidin (1:1000; #A12380, Invitrogen-Thermo Fisher Scientific) or Alexa-488 Phalloidin (1:1000; A12379, Invitrogen-Thermo Fisher Scientific) to stain F-actin, for 1 hour at room temperature. Next, coverslips were washed with 1x PBST twice for 5 minutes each. Then, coverslips were stained with DAPI (BD Biosciences, # 564907, RRID:AB_2869624) for 5 minutes, washed once with PBS and mounted on glass slides (#N/A141, ACADEMY) using Aquamount mounting medium (#14-390-5, Thermo Fisher Scientific).

Cells were imaged on a Leica DMi8 microscope with a 20x objective (HC PL FLUOTAR 20x/0.75) or a 63x air objective (HC PL FLUOTAR 63x/0.70) equipped with a Leica DFC9000GT camera, or in a Leica TCS SP8 SMD laser scanning microscope equipped with a EL6000 Fl light source, FOV scanner SP8, - 3.6 kHz tuneable speed, 60x water objective (HC PL APO CS2 20x/0.75). SP8 images were captured as stacks (11 planes, 0.5 μm steps) in conventional confocal or lightning mode which is based on adaptive image reconstruction^65^ at 1-10% laser power using photomultiplier tube (PTM) detectors in sequential mode at 488 and 568 nm, and Pinhole: 1.00 AU, in a LAS X Life Science Microscope Software (version 3.5.5.19976, RRID:SCR_013673).

#### Flow cytometry assays

For flow cytometry DNA quantification, 5 × 10^6^–2 × 10^7^ HCT116 stably expressing APC WT or m4 cells were collected, spun at 10,000*g* and fixed with 1 ml of 70% ice-cold ethanol for 30 min at 4 °C. After two washes with PBS, cells were resuspended in 50ul of preboiled RNAse solution (100 ug/ml; #70856-3, Merck) and 200 ul of propidium iodide solution (50 μg/ml; #25535-16-4, Merck), vortexed and incubated overnight at 4 °C. The next day cells were spun at 10,000*g* and analyzed with a CytoFLEX® flow cytometer (Beckman Coulter; 405 nm laser).

#### Live-cell imaging

To investigate cell-cell junction dynamics, SW480 cells stably expressing APC-WT and APC-m4 were seeded in ibidi 35 mm^2^ dishes pre-coated with collagen I (50201; ibidi, Germany). Once confluent, cells were co-transfected with plasmids encoding E-cadherin-GFP – adherent junction marker (#280009, Addgene), and Life-Actin-mCherry (as control, #54491, Addgene). 24 hours post-transfection, medium was changed to L15 (Leibovitz, # 21083027, ThermoFisher) and cells were imaged live every 2 minutes for 20 minutes using a Leica SP8 SMD confocal laser scanning microscope equipped with a EL6000 Fl light source 60x water objective (HC PL APO CS2 20x/0.75), connected to H301-EC-LG-BL holder and Okolab stage top incubator H301-T-UNIT-BL-PLUS, Okolab CO2-UNIT-BL and HM-ACTIVE Humidity Controllers to maintain cells at 37°C with 5% CO2 and 95% humidity. Images were captured as stacks (3 z-stacks with a 0.6 μm step) using photomultiplier tube (PTM) detectors in sequential mode at 488 and 568 nm, and Pinhole: 1.00 AU, in a LAS X Life Science Microscope Software (version 3.5.5.19976).

For wound healing assays, 80,000 cells - HCT116 or SW480 APC-WT or APC-m4 - were seeded on 8-well chambers with collagen IV (Ibidi, Germany) containing DMEM medium containing high glucose buffer (Gibco, Life Technologies), 10% FBS, 20 mM L-glutamine, 1 mM sodium pyruvate, and 5 ml of Penicillin-Streptomycin antibiotics and incubated at 37°C, 5% CO_2_, and 95% humidity. Once cells were confluent, the medium was removed and replaced with fresh medium but without FBS for 6 - 7 hours. After that time, axial wounds were performed using a standard pipette tip. Then, serum-free medium was removed, and cells were washed twice with PBS (#20012027, ThermoFisher). Next, the chambers were replenished with DMEM medium containing 10% FBS. To monitor wound closure, SW480 and HCT116 cells were imaged at 30 min intervals for 24 hr or 48 hr, respectively, maintaining cells at 37°C with 5% CO_2_ and 95% humidity using an Okolab stage top incubator H301-T-UNIT-BL-PLUS, Okolab CO2-UNIT-BL and HM-ACTIVE Humidity Controllers connected to a H301-K-FRAME holder adapted and an inverted Leica DMi8 microscope (Leica microsystems). Images were captured using an HC PL FLUOTAR 10x/0.30 air objective, and a Leica DFC9000GT camera using a LAS X Life Science Microscope Software (version 3.5.5.19976). To assess repositioning of centrosomes after wounding, cells were allowed to migrate for 16 hours maintained at 37°C, 5% CO_2_ and 95% humidity in an incubator, then fixed with warm 4% paraformaldehyde in PBS and proceed for immunofluorescence to co-stain with pericentrin antibodies (centrosome marker) and DAPI (nuclear marker).

#### Imaging data analysis

For imaging data analysis, ImageJ/Fiji (v1.53k, RRID:SCR_003070/v2.3, RRID:SCR_002285), python (v2.12, RRID:SCR_008394), Microsoft Excel (v16, RRID:SCR_016137), GraphPad Prism (v9, RRID:SCR_002798) and Adobe Illustrator (v26.5, RRID:SCR_010279) were used.

##### Fluorescence intensity measurements

To measure APC protein levels in cells, a custom macro in Fiji was used to generate and save sum slices z-projections for all images. Next, cell boundaries were manually drawn on these images and saved in a separate folder. Another custom macro in Fiji was then used to measure integrated density, mean grey value, and cell area. Finally, corrected total cell fluorescence (CTCF) was calculated using the following formula:

CTCF = Integrated density – (Area of selected cell X Mean fluorescence of background readings)

To measure F-Actin and Occludin protein levels at the cell junctions, immunofluorescence images were opened in Fiji and cell boundaries were manually traced from the F-Actin channel. Regions of interest (ROIs) from F-Actin channel were saved and over imposed with cells/junctions in the Occludin channel. Mean grey values were obtained in ImageJ for both channels and saved as .CSV. Data was then analyzed and plotted in GraphPad Prism (version 9.0; GraphPad Software, La Jolla, CA) as Superplots.^66^^,^^67^ Superplots displayed the mean of the different replicates (N = 4 in these cases, in circles) and the distribution of ‘n’ as level of F-Actin or Occludin at the cell junctions (as color-code dots) was superimposed to the means as violin plot. Mean was shown as a line; paired two-tailed t test was performed to find the statistical differences between replicates (N = 4). This way of representing data linked paired measurements together and conveyed the repeatability of the work, eliminating the need to normalize data to directly compare different experimental replicas.

##### Qualitative assessment of cell junction appearance

To qualitatively assess the effect on cell-cell junctions of APC-driven actin nucleation impairment, a criterion based on both the healthiness and morphology of the cell junction of each cell was established. We divided cells in three different categories: ‘*normal’, ‘discontinuous’*, and *‘round’*. *Normal* cells were defined as cells with all, or at least three cell junctions labelled with F-actin throughout the cell junction (i.e. continuous signal), whereas *discontinuous* cells were those cells with two or more disrupted cell junctions, therefore not all cell junctions were properly labeled. ‘*Round’* cells were defined as cells that have lost their typical morphology and acquired roughly a circular shape. Mitotic cells were excluded from the measurements. Qualitative count was performed in a blind way by several members of the team for three different sets of images. The percentage of cells corresponding to each category was calculated using Microsoft Excel, and then plotted with GraphPad Prism (version 9.0; GraphPad Software, La Jolla, CA).

##### Cell size quantification

For cell size quantification of individual cells in monolayers, we first generated TIFF files of the middle frame of F-actin immunofluorescence images using Fiji. Then, individual cell size was quantified using a deep-learning-based segmentation method called CellPose (^40^, RRID:SCR_021716). Briefly, parameters to segment cells were chosen in function of the cell diameter, which we estimated using Fiji. Images were loaded into CellPose and “CP” was selected as model to segment cells. The outlines of the cells were saved as .txt. Original TIFF files were opened in Fiji, the macro called imagej_roi_converter.py was run and outlines were over imposed on the monolayer image. For accurateness of the results, cells from the edges of the images were manually scrutinized and removed from the counts. We measured cell areas which were saved as .CSV. Data was then analyzed and plotted with GraphPad Prism (version 9.0; GraphPad Software, La Jolla, CA).

#### Flow cytometry DNA content analysis

Initially, cell cycle phase distribution was obtained in a CytoFLEX® flow cytometer (Beckman Coulter; 405 nm laser). Next, cells were gated by size and granularity on forward scatter (FSC)/side scatter (SSC) plot and cell debris was excluded from the analysis. Mean fluorescence intensity from 10,000 cells/events was scored for each sample. Further quantitative data analyses to gate out debris were performed using the FlowJo™ software version 10.8.1 (BD Life Sciences), a software application with an integrated environment for viewing and analyzing flow cytometric data. Plots were generated in GraphPad Prism.

##### Cell migration analysis

Individual cells migrating at the edge of the wound were tracked using the manual tracking from ImageJ, whose coordinates were saved as a .txt file. Those files were then exported into Chemotaxis and Migration Tool version 2.0 software (Ibidi, Germany RRID: SCR_022708) to obtain migratory related parameters such as the directionality, speed [μm/min], Euclidean Distance [μm], and Accumulated Distance [μm] of each cell tracked Data were combined from time-lapse image series collected from at least three independent experimental days, then plotted with GraphPad Prism (version 9.0; GraphPad Software, La Jolla, CA).

##### Cell junction analysis

To measure cell junction length, a line was manually drawn on the cell junction using the “segmented line” tool in Fiji and line length was measured using the “measure” tool. To calculate the junction angle, a straight line was drawn joining the two vertexes and the line angle was measured using the “measure” tool. The minimum angle was termed angle 0 and the rest were normalized by subtracting the minimum angle. Junction vertex disruption was qualitatively analyzed. Junctions with none, one or both vertexes disrupted were labelled as 0, 1 and 2 vertexes disrupted, respectively.

To measure junction velocities, kymographs were generated by drawing a straight line perpendicular to the junction using the “multi-kymograph” tool in Fiji. Next, the “segmented line” tool was used to trace the junction by connecting maximum pixel intensity in each time frame. To measure vertex velocities: kymographs were generated by drawing a straight line along the junction joining the two vertexes using the “multi-kymograph” tool in Fiji. Next, the “segmented line” tool was used to trace each vertex by connecting the maximum pixel intensity on each side of the junction in each time frame. Finally, velocities were calculated by measuring the length of the segmented line using the “measure” tool and dividing it by time. All data was plotted in GraphPad Prism as Superplots.^66^^,^^67^ Superplots displayed the mean of the different replicates (in circles, N = 3) and the distribution of ‘n’ as cell junctions or vertexes (as stated, as color-code dots) was superimposed to the means as violin plot. Mean and standard deviation were shown as lines; paired (or ratio paired when fold change was compared) two-tailed t test was performed to find the statistical differences between replicates (N = 3 in all panels). This way of representing data linked paired measurements together and conveyed the repeatability of the work, eliminating the need to normalize data to directly compare different experimental replicas.

#### Centrosome reorientation analysis

Centrosome reorientation was determined from images at 16 hours after wounding of APC-WT and APC-m4 monolayers, fixed and stained with anti-pericentrin (centrosome) and DAPI (nucleus). Pericentrin signal was used to measure orientation of the centrosome (angles, ‘x°’ in degrees). Angles were scored from 0-180° by drawing two lines on the cells, one parallel to the wound splitting the nucleus in two halves (front and rear) and the other one, perpendicular to the front of the wounding migrating cells. When pericentrin signal was at the rear of the nucleus, the angle was calculated as 180°- x°. Mitotic cells were not considered for this analysis. By contrast, when signal was at the front, the angle was kept as x°. Angles were plotted as heat maps in GraphPad Prism. The color gradient shows the different angles with intensities ranging from 0-180 grades (in degrees) that correspond to less oriented (yellow) to more oriented (purple), respectively.

#### Scanning Electron Microscopy

To study the ultrastructure of the cell junctions, 200000 HCT116 cells were seeded on collagen-precoated (Advanced BioMatrix - PureCol® Solution, 3 mg/ml (bovine) #5005, AdvanceBiomatrix) 18 mm^2^ round coverslips. After cells grew to confluency (approximately 24 hours), cells were fixed with 2.5% glutaraldehyde (Polyscience, Cat# 01909-10) in 0.1M Sodium Phosphate buffer pH 7.2 for 3 hours. Samples were then washed three times with 0.1mM Sodium Phosphate buffer pH 7.2 for 10 minutes. Next, samples were post-fixed in 2% Osmium tetroxide (OsO4, # O5500-250MG, Sigma-Aldrich) in buffer and left on ice for one hour. A second round of three 10-minutes wash in 0.1mM Sodium Phosphate buffer pH 7.2 was performed. After that, samples were fully dehydrated in a graded series of ethanol solutions (10 minutes per solution), followed by hexamethyldisilazane (HMDS, Merck Life Science Ltd, Cat# 804324) twice for 10 minutes. Samples were left at room temperature overnight to ensure full evaporation of HMDS. Finally, samples were mounted and sputtered coat with gold and Palladium using a SC7640 sputter coater, before observation using a Jeol 6490 scanning electron microscope operating at 5kV.

### Quantification and statistical analysis

All experiments were repeated multiple times, as indicated in figure legends. Data were pooled and, if required, analyzed further in Microsoft Excel (v16), and plotted in GraphPad Prism (v9.0; GraphPad Software, La Jolla, CA). Figure legends specify the n, errors, and the statistical test used. Data distributions were tested for normality using the D'Agostino-Pearson omnibus normality test, and statistical differences among conditions were calculated using ordinary one-way or two-way ANOVA Holm-Sidak or Tukey multiple comparisons tests, Mann-Whitney U test (non-parametric) or unpaired t-test with Welch’s correction (parametric) in GraphPad Prism (v9.0; GraphPad Software, La Jolla, CA). For Figures 1 and 2, Superplots were displayed as in Lord et al.^66^ and Kenny and Schoen^67^, displaying the mean of the different replicates and superimposed the distribution of ‘n’ (as color-code dots) as violin plot. Mean from the different replicates (but not from the total ‘n’), standard deviation and paired two-tailed t test were used to find the statistical differences between replicates. This way of representing data linked paired measurements together and conveyed the repeatability of the work, eliminating the need to normalize data to directly compare different experimental replicas. Differences were considered significant if p-value was <0.05 (∗), <0.01(∗∗), <0.001(∗∗∗), or < 0.0001 (∗∗∗∗), as indicated in each figure legend.

## Data Availability

• Data reported in this paper have been deposited at Zenodo (zenodo.org): https://doi.org/10.5281/zenodo.7528387 and are publicly available upon request to the lead contact, Maria Angeles Juanes. • Codes generated in this work have been deposited at Zenodo (zenodo.org): https://doi.org/10.5281/zenodo.7528387 and are publicly available upon request to the lead contact. • Any additional information required to reanalyze the data reported in this paper is available from the lead contact upon request.
