# Supplementary material for: APC-driven actin nucleation powers collective cell dynamics in colorectal cancer cells
Source: iScience. 2023 Apr 6;26(5):106583. doi: 10.1016/j.isci.2023.106583 (PMC10148130; doi:10.1016/j.isci.2023.106583)
Supplement: Document S1. Figures S1 and S2 [file mmc1.pdf]

iScience, Volume *26*

Supplemental information

APC-driven actin nucleation powers collective cell dynamics in colorectal cancer cells

Lautaro Baro, Asifa Islam, Hannah M. Brown, Zoë A. Bell, and M. Angeles Juanes

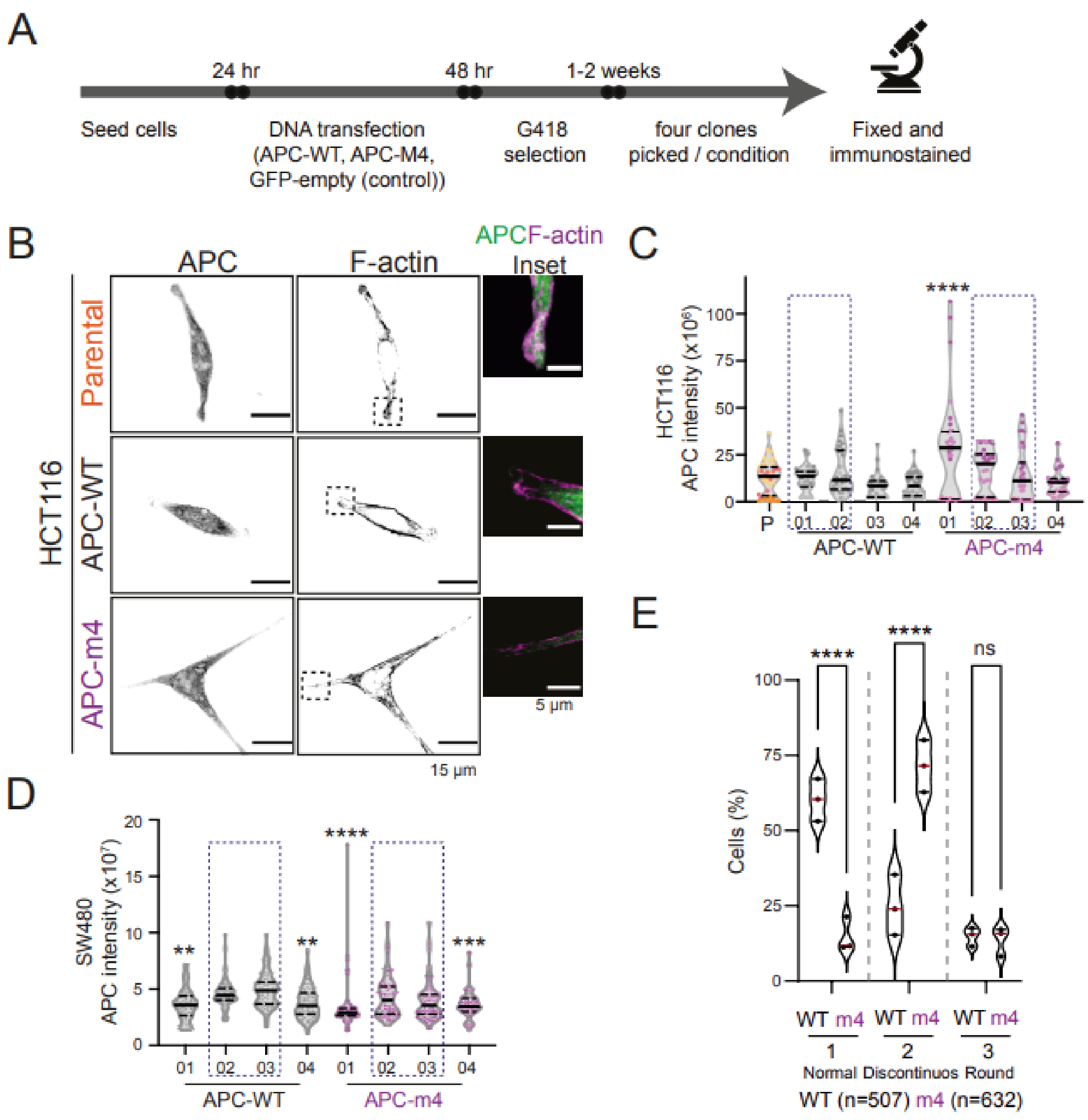

Figure S1. Related to Figure 1. Generation of stable colorectal cancer cell lines and cell junction integrity effects in APC-m4 mutant cells. (A) Experiment regimen. **(B)** Representative immunofluorescence images of HCT116 colorectal cancer (parental, and stably expressing stably either APC-WT or APC-m4) cells treated as in (A) showing APC and F-actin protein signals. Scale bar = 15 µm, inset = 5 µm. **(C)** Violin plot showing APC signal in HCT116 colorectal cancer (parental, and stably expressing either APC-WT or APC-m4) cells, and from the corresponding clones (C01-04). n(parental) = 37, n(APC-WT:C01) = 29, n(APC-WT:C02) = 33, n(APC-WT:C03) = 31, n(APC-WT:C04) = 35, n(APC-m4:C01) = 32, n(APC-m4:C02) = 30, n(APC-m4:C03) = 35 and n(APC-m4:C04) = 25. **(D)** Violin plot showing APC signal of SW480 APC-WT and APC-m4 stably expressing cells, and from the corresponding clones (C01-04). n(APC-WT:C01) = 83, n(APC-WT:C02) = 94, n(APC-WT:C03) = 86, n(APC-WT:C04) = 95, n(APC-m4:C01) = 94, n(APC-m4:C02) = 87, n(APC-m4:C03) = 96 and n(APC-m4:C04) = 92. Data in panels (C-D) are from two independent repeats. The solid line is median and dotted lines are quartiles. One-way ANOVA with Tukey correction was performed to find the statistical differences. '*' is $p<0.05$, '**' is $p<0.01$, '***' is $p<0.001$ and '****' is $p<0.0001$. **(E)** Qualitative graph showing the percentage of HCT116 APC-WT or APC-m4 cells that belongs to the "normal", "discontinuous", and "round" category. Two-way ANOVA Sidak's multiple comparisons test was performed. '****' is $p<0.0001$, and 'ns' is not significant. n(APC-WT) = 507, n(APC-m4) = 632. Data were obtained from three independent repeats.

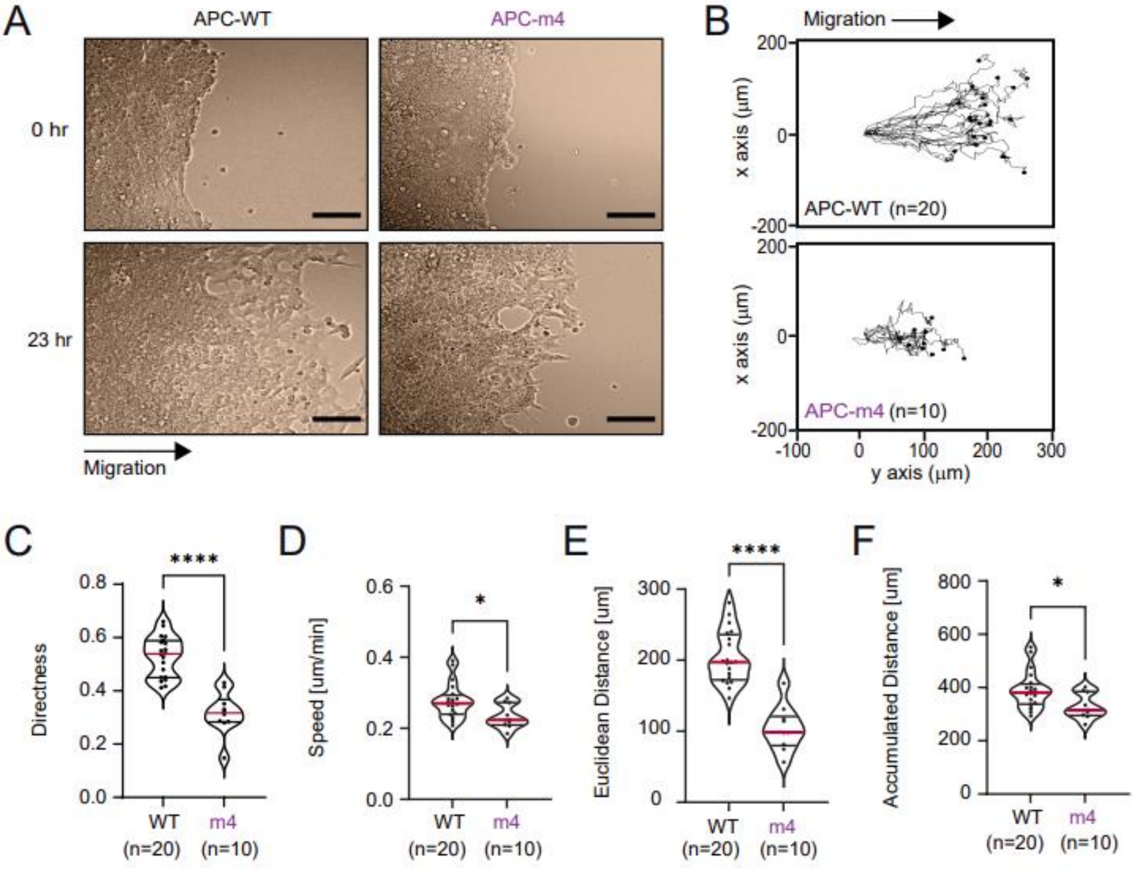

Figure S2. Related to Figure 4. Expression of APC-m4 mutant causes defects in directionality and speed of colorectal cancer wounded monolayers. All data are from SW480 cells stably expressing APC-WT or APC-m4. **(A)** Representative wound healing assays showing cells at 0 and 23 hours after scratch. The arrow indicates the direction of the migration front. Scale bar: 100 µm. **(B)** Representative traces of the migration paths of individual cells, moving into a wound site, displayed in horizontal arrays. Black arrow indicates the direction of the migrating cells. **(C-E)** Violin plot showing the directionality (B), velocity (C), Euclidean distance (D), and accumulated distance (E) of individual cells from the edge of the wound during a 23-hour observation window. The red line is the median and the black lines are the quartiles. Mann-Whitney U-test was performed to find the statistical differences. '*' is $p<0.05$ and '****' is $p<0.0001$. Data in panels C to F are from three independent repeats. In panels B to F, n(APC-WT) = 20, n(APC-m4) = 10.
